# Reply: Limitations in the creation of an automatic diagnosis tool for dysgraphia

**DOI:** 10.1038/s41746-019-0115-z

**Published:** 2019-05-09

**Authors:** Thibault Asselborn, Thomas Gargot, Łukasz Kidziński, Wafa Johal, David Cohen, Caroline Jolly, Pierre Dillenbourg

**Affiliations:** 1 CHILI lab, EPFL, Route Cantonale, 1015 Lausanne, Switzerland; 2 Psychiatrie de l’Enfant et de l’Adolescent, Pitié Salpêtriére - Charles Foix, Assistance Publique Hôpitaux de Paris, 47/83 boulevard de l’Hôpital, 75013 Paris, France; 3 ISIR, Sorbonne Université, 4 Place Jussieu, 75005 Paris, France; 4 CHART Laboratory - EA 4004, TIM, Paris 8 University, 93526 Saint Denis, France; 5 Department of Bioengineering, Stanford University, 443 Via Ortega, Stanford, CA 94305 USA; 6 LSRO Lab, EPFL, Route Cantonale, 1015 Lausanne, Switzerland; 7 LPNC, Univ. Grenoble Alpes, F-38040, Grenoble - CNRS, LPNC UMR 5105, F-38040 Grenoble, France

**Keywords:** Diagnosis, Disability

We thank Labyt et al. for their comments about methodological limitations of our paper.^1^ We carefully revisited the paper and the data, taking into account the critical perspective of the authors. We attempted to further explain raisen issues. However, if things remain unclear, we are ready to perform more analysis and provide more details.

Concerning the tablet calibration: Ideally the same tablet could have been used for the data collection of the two datasets. As it was not the case, we quantified their difference in pressure acquisition. Following the advice of the tablet manufacturer (Wacom Co., Ltd), a pressure calibration was performed on the two tablets. A construction was made to position the pen vertically on the surface of the tablets with minimal friction. Fifteen different weights (called X) from 0 g (pen without load) to 400 g (saturation of both tablets)) were used as an input while the values returned by each tablets (called Y) were logged. We then extracted the relation X/Y for the two tablets which ended up being very similar (the Spearman correlation shows a correlation of 0.9915 (*p* = 5.32e-12), mean square error of 0.6). A 4th degree polynomial fit was created to model the function describing the X/Y relation of the first tablet and used on the input of the second in order to rectify its output. After this correction, the Spearman correlation was found to be 99.998% (*p* = 1.81e-21) and the mean squared error was 5.1e-3.

Concerning the in the in-air-time feature: As authors correctly spotted, we didn’t include correction to take care of the irregularities highlighted by Labyt et al. in the acquisition of the data of the D dataset. Note however, that this feature was not found to be important (on the basis of the Gini importance given by the random forest) as can be seen in Table 1. After applying the correction, we retrained our model and saw that the performance of our model is not affected since the specificity and sensibility remain the same.

**Table 1:** The most important features found by the Random Forest model as well as the In Air Time Ratio feature, using Gini importance as a metric

Rank	Category	Name	Importance (Std.) [%]
1	Kinematic	Median of power spectral of speed frequencies	15.71 (9.06)
2	Kinematic	Bandwidth of speed frequencies	12.08 (8.00)
3	Pressure	Mean speed of pressure change	9.81 (6.52)
4	Static	Space between words	7.45 (6.73)
5	Tilt	Distance to mean of speed of tilt-X change frequencies	6.07 (4.30)
6	Kinematic	Distance to mean of speed change frequencies	5.18 (4.73)
7	Tilt	Bandwidth of speed of tilt-X change frequencies	4.10 (4.64)
8	Tilt	Median of power spectral of tilt-Y change frequencies	2.97 (3.33)
…	…	…	…
16	Kinematic	In Air Time Ratio	0.91 (0.86)

We report the ranks, features categories and their importance averaged for the 25 folds and the standard deviation of importance over all folds

Concerning the handwriting evolution according to the age: We are obviously aware that handwriting evolves with age. In the same way, handwriting evolves differently according to the gender of the child or if he/she is left-handed or right-handed. To account for these variabilities, we included these features (age, gender, and laterality) in the model. The model should then be more robust to these demographic features.

Concerning the recruitment limitations: The people in charge of giving the test to the children were also different, as well as the conditions in which the children passed the test. The protocol was very simple and standardized: children just had to write the BHK text on a blank sheet of paper located on top of a digital tablet for 5 min. In this situation, we believe it is fair to assume that the variability of examiners is negligible.

In addition, Labyt et al. argue that our D dataset may not be representative of the general dysgraphic population leading the model to be biased toward more severe cases of dysgraphia.

If we wanted a D dataset representative of the general dysgraphic population and thus free of bias, the dysgraphic children should have been recruited from the same schools where the tests were conducted for the TD dataset. Since only 5% of children present dysgraphia in the general population, we would have needed to recruit more than a thousand children to reach the number of dysgraphic children we have in our study. It is for this reason that we recruited the D dataset in special centers for this study.

In our future work, we aim to collect more data from schools and therapy centers in order to predict with higher accuracy for the whole spectrum of dysgraphic children.

Concerning the following issue: some of the TD children might be dysgraphic, our results are thus too good: We are also aware of this problem, and that is why we wrote the following sentence in the discussion section to show the current limitation of our model: Note that the inter-rater correlation in BHK is 0.89. Since our algorithm outperforms this value, we conclude that the algorithm learned to mimic the rater. These findings suggest that adding data from other raters should not only reduce bias, but also allow us to surpass the accuracy of each individual rater.

Besides, as mentioned in the paper, we are in the process of having all the 296 tests of TD children as well as the 56 dysgraphic children from the D dataset by three rater experts to be the most objective possible. This new annotated data will help us explore the performance of the model in its current stage. Secondly, it will allow us to retrain our model with strictly controlled D and TD datasets that will help the model to better detect dysgraphia. Following the argument of Labyt et al. that dysgraphic children from school present less severe dysgaphia compared to the ones from the Reference Center for Language and Learning Disorders of the Grenoble Hospital (Centre Referent des Troubles du Langage et des Apprentissages, CRTLA, Centre-Hospitalier-Universitaire Grenoble), adding handwriting of dysgraphic children recruited from school in our D dataset will help the model to reduce its bias towards severe dysgraphia.

We acknowledge that the presence of false positive in our classification of TD children could have been more developed in the discussion but we were constrained by the page-limit and chose to discuss other points.

Our new study on the annotated data will give more insights on this particular point.

To conclude, we refute the term of errors, since Labyt et al. are not showing errors in our paper but are questioning some of the approaches that we decided to follow. We believe that our work will be improved with the proper annotation of the D and TD dataset. Finally, we think that like any innovative new claim in medicine, it would be necessary to replicate the study. That is why we would like to see other scientists work on the same topic in order to gain better knowledge on graphomotor disabilities.

## Data Availability

As it is possible to identify participants from the data, ethical requirements do not permit us to share participant data from this study.

## Code availability

Code is available from the corresponding author upon reasonable request.
